# Leveraging Kaizen with Process Mining in Healthcare Settings: A Conceptual Framework for Data-Driven Continuous Improvement

**DOI:** 10.3390/healthcare13080941

**Published:** 2025-04-19

**Authors:** Mohammad Najeh Samara, Kimberly D. Harry

**Affiliations:** School of Systems Science and Industrial Engineering, Binghamton University, Binghamton, NY 13902, USA

**Keywords:** Kaizen, process mining, healthcare process improvement, integrated framework, continuous improvement

## Abstract

**Background/Objectives:** Healthcare systems face persistent challenges in improving efficiency, optimizing resources, and delivering high-quality care. Traditional continuous improvement methodologies often rely on subjective assessments, while data-driven approaches typically lack human-centered adaptability. This study aims to develop an integrated framework combining Kaizen principles with Process Mining capabilities to address these limitations in healthcare process optimization. **Methods:** This research employed a structured literature review approach to identify key concepts, methodologies, and applications of both Kaizen and Process Mining in healthcare settings. The study synthesized insights from the peer-reviewed literature published in the last two decades to develop a conceptual framework integrating these approaches for healthcare process improvement. **Results:** The proposed framework combines Kaizen’s employee-driven approach to eliminating inefficiencies with Process Mining’s ability to analyze workflow data and identify process deviations. The integration is structured into four key phases: data collection, process analysis, Kaizen events, and continuous monitoring. This structure creates a feedback loop where data-driven insights inform collaborative problem-solving, resulting in sustained improvements validated through objective process analysis. **Conclusions:** The integration of Kaizen and Process Mining offers a promising approach to enhancing workflow efficiency, reducing operational errors, and improving resource utilization in healthcare settings. While challenges such as data quality concerns, resource constraints, and potential resistance to change must be addressed, the framework provides a foundation for more effective process optimization. Future research should focus on empirical validation, AI-enhanced analytics, and assessing adaptability across diverse healthcare contexts.

## 1. Introduction

Kaizen, which is derived from the Japanese term meaning “continuous improvement”, is a systematic approach based on the philosophy of achieving incremental changes to processes that collectively result in significant improvements [1]. Although it was initially developed within the manufacturing sector, particularly in Toyota’s Production System (TPS), it has been widely adopted in healthcare to address operational inefficiencies and improve patient care. Its core principles include the elimination of waste, the enhancement of efficiency, and the active involvement of all staff, including clinicians, administrators, and frontline employees [2,3]. In healthcare, Kaizen promotes a culture where problems such as delayed patient discharges, medication errors, and inefficiencies in appointment scheduling are regularly identified and resolved [4]. Tools such as process mapping, brainstorming, Plan-Do-Study-Act (PDSA), and the Plan-Do-Check-Act (PDCA) cycle are commonly utilized to streamline workflows and enhance patient outcomes [5]. Kaizen provides a structured methodology for translating its philosophy into actionable steps that drive tangible improvements. For example, a Kaizen event (KE, also commonly referred to as a Kaizen workshop in healthcare settings), an accelerated, team-based Kaizen improvement tool in a hospital might begin by identifying inefficiencies in the emergency room admission process, followed by assembling a cross-functional team to analyze bottlenecks and propose solutions [6]. Moreover, a culture of collaboration and innovation is fostered, as healthcare staff are encouraged to actively participate in the improvement process [7]. Consequently, Kaizen has been recognized as an essential methodology for improving healthcare delivery and aligning operational outcomes with patient-centered goals.

Traditional Kaizen methods in healthcare often depend on the expertise and intuition of participants, such as physicians, nurses, frontline workers, and administrators, to identify inefficiencies and propose solutions [8]. This reliance can lead to incomplete or inaccurate outcomes, especially in large, data-intensive systems like hospital networks [8]. For example, although KEs have been successfully applied across various industries, they possess notable limitations. They often rely heavily on subjective observations and manual evaluations, which can lead to inconsistencies and a lack of objectivity [9]. Additionally, manual assessments during KEs are time-consuming, slowing the pace of change in high-pressure environments like intensive care units or emergency departments [3]. Traditional Kaizen approaches also struggle to manage dynamic and complex healthcare processes, where patient needs, treatment protocols, and regulatory requirements frequently change. As a result, proposed improvements may address surface-level issues rather than the systemic root causes of inefficiencies [10]. Furthermore, traditional Kaizen lacks the predictive capabilities essential for anticipating potential problems, such as surges in patient volume or adverse and disruptive clinical events [11]. These challenges have led to a growing interest in incorporating data-driven methodologies to improve process improvement initiatives in healthcare. PM has emerged as a valuable analytical tool, offering actionable insights based on objective data rather than subjective judgment [12]. In healthcare, PM utilizes event logs from electronic health records (EHRs) [13], hospital information systems, and other digital platforms to uncover actual workflows, such as patient admission and discharge processes [14], lab testing timelines, and surgical workflows [15].

Healthcare systems face unique process improvement challenges, including resource limitations [16,17], complexities in interdisciplinary coordination [18,19], and high-stakes environments where errors can have serious consequences [20,21]. Despite Kaizen’s benefits, its traditional implementation in healthcare often lacks objective measurement tools to validate improvements [22]. Conversely, while data-driven, PM typically operates without the cultural and engagement elements essential for sustainable change [23]. This research addresses the critical gap between these approaches by proposing an integrated framework that combines Kaizen’s human-centered methodology with PM’s analytical capabilities.

The primary objective of this work is to propose a conceptual framework that integrates PM with Kaizen approaches, offering a theoretical foundation aimed at enhancing continuous improvement practices in a transformative way. This framework combines the structured methodology of Kaizen with the data-driven insights provided by PM, addressing the limitations of traditional approaches such as reliance on subjective observations and static analysis. A novel methodology is introduced to conceptualize how these two approaches can merge effectively to improve process optimization, root cause identification, and adaptability in continuous improvement initiatives within hospital settings. Furthermore, the study critically examines and highlights the theoretical advantages of the integrated approach over traditional methods, emphasizing enhanced objectivity, improved alignment with complex and dynamic processes, and greater scalability. This study provides a novel theoretical contribution to the academic discourse on continuous improvement methodologies, establishing a foundation for future empirical research and practical applications. To the best of the authors’ knowledge, this is the first study to develop a conceptual framework that integrates Kaizen and PM in healthcare through a structured and comprehensive approach. This integration bridges the gap between traditional qualitative improvement strategies and modern data-driven methodologies, addressing the key limitations of existing process optimization techniques.

The paper is structured as follows: Section 2 presents the methodological approach, detailing the selection criteria for the literature, data sources, and the framework development process. Section 3 outlines the theoretical background, covering the fundamental principles of Kaizen and PM, along with their applications in healthcare settings. Section 4 introduces the proposed conceptual framework, explaining its key components and stepwise implementation. Section 5 explores validation strategies, including potential pilot studies and simulation-based assessments. Section 6 acknowledges the limitations and proposes directions for future research. Finally, the paper concludes with a summary of the key findings and recommendations for further study.

## 2. Methodological Approach

This study employs a systematic search and review approach, as described by Grant and Booth [24], combining the strengths of a critical review with a comprehensive search process. Unlike purely systematic reviews that address narrow questions with prescribed methods [25], this approach enables us to address the broader conceptual question of how Kaizen and PM might be integrated while maintaining methodological rigor. Following the best practices in conceptual model development [26], a targeted strategy was used to identify the most relevant and methodologically sound sources in the fields of process improvement and data-driven healthcare optimization. The review process emphasized key theoretical foundations, methodological advancements, and practical applications to ensure a comprehensive yet adaptable synthesis of existing research. This structured methodology enhances the framework’s theoretical rigor, practical relevance, and applicability in healthcare settings by aligning academic discourse with real-world implementation challenges [27].

### 2.1. Selection Criteria

To establish a strong theoretical foundation, relevant studies and articles were selected based on the following criteria:

1. **Kaizen in Healthcare:** Research focusing on the implementation of Kaizen methodologies in hospital operations, process optimization, and continuous improvement initiatives;
2. **PM Applications**: Studies examining how PM has been applied in healthcare, particularly in workflow optimization, bottleneck identification, and compliance monitoring;
3. **Integrated Improvement Models:** Articles exploring data-driven approaches in Lean healthcare and case studies where data analytics supported process improvement;
4. **Recent and High-Quality Sources:** Peer-reviewed journal articles, case studies, and authoritative healthcare management reports published within the last 20 years.

### 2.2. Databases and Search Strategy

A multi-database search strategy was employed to ensure comprehensive coverage of the existing research:

1. **Google Scholar**—Searched for broad academic discussions on Kaizen, Process Mining, and healthcare process improvement;
2. **PubMed**—Focused on healthcare applications of Lean and data-driven methodologies;
3. **IEEE Xplore and SpringerLink**—Identified relevant studies on PM algorithms and their real-world applications;
4. **ProQuest and ScienceDirect**—Sourced peer-reviewed case studies and empirical research on healthcare process optimization.

Search terms included “Kaizen in healthcare”, “Process Mining for hospital workflows”, “Lean healthcare process optimization”, and “data-driven continuous improvement in hospitals”.

Following a structured methodology for systematic search and review, this study implemented a comprehensive three-stage process:

**1. Search:** Systematic searches were conducted across the selected databases using predefined search strings that combined terms such as “Kaizen”, “Process Mining”, “healthcare”, “continuous improvement”, and “data-driven”. These searches were supplemented by citation tracking (both forward and backward) of key articles. Search strings were adapted to each database’s syntax while maintaining conceptual consistency. The search was limited to English-language publications from 2000 to 2024 to ensure contemporary relevance.

Articles were selected based on their conceptual contribution to either Kaizen principles or PM applications in healthcare settings, with particular emphasis on those that offered insights into potential integration points. Quality assessment focused on methodological coherence, conceptual clarity, and applicability to healthcare contexts rather than adherence to a formal grading system, which aligns with the interpretative rather than aggregative nature of our review;

**2. Appraisal:** Retrieved citations underwent initial screening based on the title and abstract to assess alignment with the inclusion criteria. Although formal quality assessment tools were not applied, full-text studies were evaluated based on the following criteria: (1) methodological clarity and appropriateness, (2) relevance to healthcare settings, (3) conceptual contribution to either Kaizen or PM, and (4) applicability to integration efforts. Studies were purposefully prioritized for inclusion during the screening and appraisal process in accordance with the criteria mentioned above;

**3. Synthesis:** Data extraction was performed using a standardized template to capture the key constructs, methodologies, contexts, findings, and limitations of each study. Thematic synthesis was then used to identify complementary elements between the Kaizen and PM approaches. This was further supported by concept mapping to visualize the potential integration points.

### 2.3. Analysis and Model Development

The study followed a structured approach to identify key insights and integrate them into the proposed framework:

1. **Identification of Key Concepts**—The literature was analyzed to extract the fundamental principles of Kaizen and PM;
2. **Comparative Analysis**—A theoretical evaluation was conducted to compare traditional Kaizen approaches with PM-based methods;
3. **Framework Development**—Insights were synthesized to design a conceptual model that integrates Kaizen and PM, ensuring synergy between employee-driven problem-solving and data-driven process optimization.

Our analysis and framework development followed an interpretative rather than aggregative approach, aimed at conceptual innovation rather than merely summarizing the existing knowledge. Through iterative refinement and validation against the literature, we developed an integrated conceptual model that preserves the essential characteristics of both approaches while addressing their individual limitations, then critically examined the proposed framework against established healthcare process improvement models to identify potential advantages, limitations, and implementation considerations.

## 3. Theoretical Background

In this section, we examine the foundational theories underpinning our study, analyzing the principles of the traditional Kaizen methodology and the fundamentals of PM. This exploration sets the stage for our proposed integration model, which aims to enhance data-driven continuous improvement in organizational processes.

### 3.1. Traditional Kaizen Methodology in Healthcare Settings

The Kaizen methodology in healthcare represents a structured approach to continuous improvement characterized by specific philosophical principles, implementation mechanisms, and demonstrated outcomes. At its foundation, Kaizen emphasizes the principle of “doing better every day, with everyone, and everywhere”, fostering collaboration, innovation, and the elimination of inefficiencies [28].

#### 3.1.1. Conceptual Framework and Implementation Approaches

Analysis of the literature reveals a consistent conceptual framework with three distinct but complementary implementation approaches in healthcare: (1) hard practice-oriented approaches focusing on technical outcomes like process optimization and waste reduction; (2) soft practice-oriented approaches prioritizing social outcomes such as improved communication and employee morale; and (3) full-Lean adherence combining both dimensions for comprehensive improvements [11]. These approaches are operationalized through a well-defined toolkit, including value stream mapping (VSM), Kaizen events (KEs), 5S methodology, and Pareto analysis to identify and address inefficiencies [29].

The implementation mechanisms span a continuum from structured, large-scale methodologies such as FOCUS-PDSA cycles to smaller, frontline-driven initiatives like Teian (“Suggestion”) systems [28]. This range of mechanisms enables adaptability across different healthcare contexts while maintaining the core philosophical principles. Regardless of scale, successful implementations consistently demonstrate dual benefits: technical outcomes (efficiency improvements, error reduction) and social benefits (enhanced team engagement, higher morale) [11,28,30].

#### 3.1.2. Critical Success Factors and Barriers

The synthesis of implementation studies identifies several critical success factors for Kaizen in healthcare. Leadership engagement and organizational support emerge as fundamental enablers, creating the conditions necessary for sustainable improvement [31]. Adequate resource allocation (staffing, time, infrastructure) and team cohesion consistently correlate with successful outcomes [32]. The visibility of improvements represents another key factor, with evidence showing diminished staff motivation when changes fail to produce observable effects [32].

Barriers to effective implementation form consistent patterns across healthcare settings. Resistance to change represents a primary obstacle, particularly pronounced in complex adaptive systems with established routines and hierarchical structures [28,31]. The complexity of healthcare processes, characterized by high variability and interdependence, necessitates contextual adaptations to standardized Kaizen approaches [33,34]. Resource limitations, including insufficient training opportunities and funding constraints, further challenge implementation efforts [29]. Sustainability emerges as a persistent challenge, with evidence indicating diminishing returns when structured reinforcement and ongoing engagement are absent [22].

#### 3.1.3. Adaptability and Evolutionary Patterns

The literature demonstrates Kaizen’s adaptability across diverse healthcare contexts. Implementation evidence spans from rural critical access hospitals to major academic medical centers [35,36], from clinical to administrative processes [37], and from resource-rich to resource-limited settings [38]. This adaptability extends to addressing specific pandemic-related challenges, with remote KEs emerging as a response to COVID-19 restrictions [39]. While these adaptations offered advantages in cost-effectiveness and work–life balance, they introduced new challenges related to engagement and preparation requirements, illustrating the dynamic evolution of implementation approaches.

#### 3.1.4. Outcome Patterns and Evidence Synthesis

Cross-study analysis reveals consistent patterns of improvement in process efficiency metrics, resource utilization, and safety outcomes. Multiple studies document significant reductions in process times, including laboratory turnaround times (60% reduction) [35], inpatient service times (54% reduction), emergency service times (29% reduction) [36], and chemotherapy administration times (30–33% reduction) [40]. Resource optimization similarly shows consistent improvements, with bed utilization increasing from 92% to 168% in oncology settings [36]. Safety metrics demonstrate positive patterns, with surgical errors reduced by 35% [41] and incident rates reduced by 34% [35].

The integration of Kaizen with complementary approaches represents another emerging pattern, with evidence supporting successful combinations with network systems for ICD-10 coding improvement [37] and with experiential learning techniques for safety enhancement [42]. These integrations suggest promising avenues for extending Kaizen’s effectiveness through complementary methodologies—a finding that provides theoretical support for the PM integration proposed in this paper.

### 3.2. Process Mining Fundamentals in Healthcare Settings

#### 3.2.1. Fundamentals of Process Mining

PM represents a data-driven approach to business process analysis and optimization that extracts actionable insights from event logs in information systems [43,44]. This methodology centers on three foundational elements working in concert: event logs documenting process activities, process models providing abstract representations of workflows, and analytical algorithms transforming data into meaningful insights [44,45].

The PM methodology encompasses three complementary approaches that form a comprehensive analytical framework. Process discovery generates models from event logs without prior assumptions, revealing actual workflows as they occur in practice rather than as they are assumed to function [46]. Conformance checking compares these discovered processes against predefined standards or expectations, identifying the deviations and compliance issues critical for regulatory adherence and error detection [46,47]. Process enhancement builds on these insights to implement targeted improvements by addressing inefficiencies, optimizing resource allocation, and streamlining workflows [46,48].

Each of these approaches employs specialized techniques designed to address specific analytical needs. Process discovery algorithms transform raw event data into structured visual representations that capture process complexity while maintaining interpretability [45]. Conformance checking methods identify and quantify discrepancies between actual and expected processes, enabling real-time monitoring and proactive intervention [46]. Enhancement techniques translate analytical findings into practical improvements through bottleneck elimination, resource reallocation, and workflow standardization [48].

Recent developments in PM focus on increasing standardization, expanding application domains, and enhancing data quality. Methodological standardization efforts aim to improve experimental design consistency and result comparability across studies [49,50]. As applications expand beyond traditional sectors, PM is increasingly recognized for its potential to optimize resource utilization and minimize waste across diverse organizational contexts [48].

#### 3.2.2. Healthcare-Specific Applications

PM has demonstrated significant value across diverse healthcare contexts, offering data-driven approaches to workflow optimization, resource utilization, and patient outcome improvement. Analysis of implementation patterns across healthcare settings reveals consistent themes in application domains, methodologies, outcomes, and challenges.

##### Application Domains and Methodological Patterns

PM applications in healthcare can be categorized into distinct domains with associated methodological approaches. Process discovery techniques predominate in outpatient settings, focusing on patient flow optimization and wait time reduction. For example, implementations have been documented in Chicago outpatient clinics [15] and Peruvian appointment systems [51]. These implementations typically employ simpler discovery algorithms to model appointment sequences and identify scheduling inefficiencies.

Inpatient care processes represent another significant application domain, with more complex methodologies being applied. Studies in Dutch gynecological oncology [52] and general inpatient management [53] employ techniques like Fuzzy Miner and Heuristics Miner to analyze multi-dimensional aspects of patient care. These approaches account for the higher complexity and variability inherent in inpatient processes, focusing on patient status transitions and departmental workload patterns.

Administrative and billing processes form a third distinct domain, characterized by conformance-focused methodologies. Hospital billing studies [54] emphasize compliance checking and variant analysis to identify deviations in claims processing and insurance verification. Alpha Miner and similar algorithms predominate in these contexts, where process standardization is critical for operational efficiency.

Specialized clinical services demonstrate the most advanced PM applications employing sophisticated techniques to model complex care pathways. Implementations in colorectal cancer screening [55], breast cancer treatment [56], and sepsis care [57] utilize Inductive Miner, semantic Process Mining, and hybrid approaches to capture treatment pathway variations while identifying opportunities for standardization and personalization.

##### Outcome Patterns and Implementation Benefits

Cross-implementation analysis reveals consistent patterns of improvement despite contextual variations. Operational metrics show significant enhancements across settings, with wait time reductions of up to 64% in outpatient contexts [51] and efficiency improvements of 98% in consultation processes. Resource optimization benefits appear consistently across domains, from improved bed utilization in oncology (92% to 168%) [36] to reduced idle resource costs in transport services [58].

Clinical outcomes similarly demonstrate consistent improvement patterns. Studies in specialized services show enhanced guideline compliance [59], more personalized treatment pathways [56], and better resource allocation during peak demand periods such as the COVID-19 pandemic [55]. Patient safety metrics improve through enhanced process visibility and standardization, with implementations documenting reduced errors and enhanced care coordination.

Visualization and management capabilities represent another consistent benefit, with PM-generated dashboards revealing unexpected workflow relationships and inefficiencies [60]. The integration of PM with EHR systems enhances the alignment between technological infrastructure and organizational workflows throughout the development lifecycle [61], addressing a critical gap in healthcare information system implementation.

##### Implementation Challenges and Mitigation Strategies

Despite these benefits, healthcare-specific challenges follow consistent patterns across implementations. Data quality and standardization emerge as primary concerns, with numerous studies [62,63] highlighting difficulties converting routine healthcare data into structured event logs suitable for analysis. The inherent variability of healthcare processes further complicates standardization efforts, particularly in clinical settings where patient needs and treatment pathways show significant variation.

Privacy and ethical considerations represent another consistent challenge area with requirements for robust anonymization and security measures to maintain compliance with regulations such as the Health Data Utilization Act [62,64]. Additional global regulations, including the Health Insurance Portability and Accountability Act (HIPAA) in the United States and the General Data Protection Regulation (GDPR) in Europe, mandate strict controls over patient data usage, requiring organizations to adopt clear consent protocols, data encryption, de-identification, and access controls [65,66]. These concerns necessitate careful protocol development addressing consent procedures and data protection measures. Institutional Review Board (IRB) oversight and regular audits can further strengthen compliance and ethical accountability [67].

Technological barriers demonstrate similar patterns across implementations. The lack of structured event logs in many healthcare systems requires specialized data pipelines to transform raw data into analyzable formats [62]. Ensuring data quality also involves implementing routine validation, cleansing steps, and error-checking mechanisms at the point of extraction [68,69]. The limited transparency of many PM tools creates challenges in healthcare where domain expertise integration is essential [63], while software selection difficulties persist due to the absence of comprehensive evaluation frameworks [55].

## 4. Proposed Integration Model

The integration of Kaizen and PM offers a transformative approach to addressing the limitations of traditional process improvement methodologies and current data-driven approaches in healthcare. The model discussed in this paper combines the structured, employee-focused principles of Kaizen with the actionable insights derived from PM, enabling organizations to responsively tackle inefficiencies, improve decision-making, and cultivate a culture of continuous improvement. This section introduces the proposed conceptual framework, highlighting its objectives, core components, implementation phases, and anticipated outcomes in healthcare systems.

### 4.1. Objectives of the Integration

The integration of Kaizen and PM combines the strengths of both methodologies while addressing their respective limitations, offering a comprehensive framework for continuous improvement in healthcare. As previously discussed, Kaizen’s structured and employee-driven approach promotes collaboration, innovation, and incremental improvements, while PM provides objective and data-driven insights into actual workflows. Together, these methodologies bridge the gap between traditional continuous improvement strategies and modern analytical tools, enabling healthcare organizations to tackle inefficiencies with greater precision and reliability. As outlined in the theoretical background, traditional Kaizen often depends on subjective observations and manual evaluations, which can lead to inconsistencies and inaccuracies in identifying process issues. At the same time, PM, while highly effective at analyzing event logs and uncovering inefficiencies, may lack the human-centered problem-solving and cultural transformation elements emphasized in Kaizen. This integration resolves these challenges by leveraging the strengths of both methods. Kaizen engages healthcare teams and fosters active participation, while PM offers insights derived from real-time data to guide interventions with accuracy and focus.

This combined framework seeks to achieve several essential outcomes. It enhances workflow efficiency through the identification and resolution of bottlenecks and delays in clinical, operational, and administrative processes. It also optimizes resource utilization by revealing inefficiencies in allocation patterns, ensuring that healthcare operations run more effectively. Decision-making is strengthened through evidence-based insights, empowering healthcare managers and staff to implement solutions aligned with organizational goals and patient needs. Ultimately, this integration fosters a culture of continuous improvement by combining the collaborative and structured problem-solving approach of Kaizen with the precision and objectivity of PM. This synergy allows organizations to address the limitations of traditional improvement methods while ensuring sustainable advancements in healthcare delivery.

### 4.2. Key Components of the Model

The proposed framework integrates the principles of Kaizen with the analytical capabilities of PM to create a comprehensive approach for continuous improvement in healthcare. This section outlines the main components of the model and explains their interaction to address inefficiencies and optimize healthcare workflows.

#### 4.2.1. Kaizen Principles

Kaizen serves as the foundation of the framework, emphasizing continuous improvement and fostering a collaborative culture in healthcare. Its key components include the following:

1. **Continuous Improvement:** A focus on making incremental changes that collectively result in significant improvements in workflows and processes [70];
2. **Active Employee Involvement:** Engaging healthcare staff at all levels, particularly frontline employees, in identifying and solving problems [71];
3. **Elimination of Inefficiencies:** Addressing waste, delays, and unnecessary steps in healthcare workflows [72];
4. **Practical Insights:** Ensuring that improvements are grounded in the day-to-day realities of healthcare operations [22];
5. **Tools and Methods:** Table 1 represents the essential tools and methods used in Kaizen to support continuous improvement in healthcare operations.

#### 4.2.2. Process Mining Tools

PM enhances Kaizen by providing data-driven insights into actual workflows. The tools employed in this model include the following:

1. **Event Logs:** Data extracted from hospital systems, such as electronic health records (EHRs), scheduling systems, and patient admission records, serve as the foundation for process analysis [64,74];
2. **Discovery Algorithms:** These algorithms generate process models from event logs, revealing the actual flow of activities and uncovering inefficiencies, delays, and deviations [49];
3. **Conformance Checking:** This technique compares discovered processes with predefined models to identify discrepancies and ensure compliance with clinical protocols and best practices [59];
4. **Enhancement Techniques:** Insights from event logs are used to refine workflows, optimize resource allocation, and improve patient care delivery [77,78].

#### 4.2.3. Interaction Between Kaizen and Process Mining

The synergy between Kaizen principles and PM tools forms the core of the proposed framework. First, PM provides healthcare teams with objective data on workflow inefficiencies, serving as the starting point for Kaizen initiatives. For example, event logs can highlight bottlenecks in emergency department operations or delays in patient discharge processes. Healthcare staff, guided by Kaizen principles, then collaborate to brainstorm solutions, implement changes, and evaluate their effectiveness through the PDCA cycle.

The integration of these methodologies creates a structured improvement process accessible to all stakeholders, regardless of their technical background:

##### Step 1: Data-Informed Problem Discovery

PM analyzes event logs from hospital systems to create visual maps of actual processes [79]—not assumed workflows, but real ones. These visualizations reveal concrete issues like unexpected waiting times, variations in care pathways, bottlenecks, and compliance deviations. For instance, PM might show that lab test results for emergency patients consistently take longer to process during shift changes, a pattern that can be identified through resource-based Process Mining techniques [80].

##### Step 2: Collaborative Root Cause Analysis

PM findings are presented in Kaizen workshops or KEs, where diverse staff collectively examine the data, fostering the engagement and ownership that are critical success factors in improvement initiatives [81]. Using tools like the “5 Whys” and fishbone diagrams, they interpret the process models from their practical experience, identify root causes, and generate potential solutions. This transforms abstract data into actionable insights based on frontline expertise.

##### Step 3: Structured Implementation

The team implements changes using Kaizen’s PDCA cycle: designing specific interventions with clear metrics, implementing them in a controlled environment, gathering feedback, and standardizing successful changes. For example, implementing a standardized handoff protocol for problematic transitions, similar to interventions that have successfully reduced communication errors and improved information exchange in perioperative settings [82].

##### Step 4: Data-Driven Validation

PM tools then monitor the modified processes to verify improvements, identify unintended consequences, and detect emerging issues [83]. This creates a feedback loop where improvements are validated by concrete data rather than subjective impressions [84].

**Figure 1:**
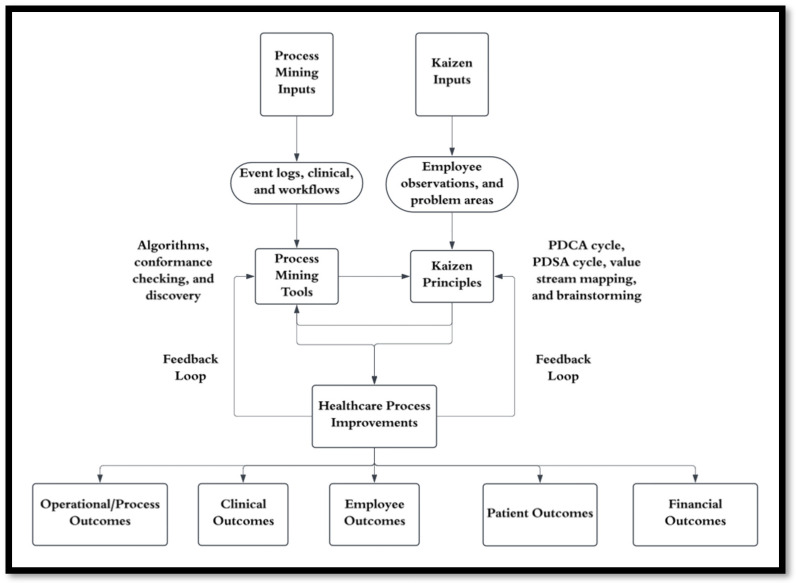
Integration of Kaizen principles and Process Mining tools in healthcare improvement. This diagram illustrates the cyclical relationship between data-driven insights from Process Mining and collaborative problem-solving through Kaizen, showing how they create a continuous feedback loop for healthcare process optimization.

Conversely, Kaizen ensures that insights generated through PM are actionable and tailored to the unique needs of the organization. By involving employees in the improvement process, Kaizen fosters a sense of ownership and commitment to change, while PM continuously monitors the outcomes of interventions, providing ongoing and expedited feedback for further refinement. Together, these components create a feedback loop where data-driven insights and collaborative problem-solving drive sustainable improvements in healthcare delivery. Figure 1 illustrates the interaction between Kaizen principles and PM tools, highlighting how their integration creates a continuous feedback loop for improving healthcare processes. (See Supplementary Materials: Figure S1 for a detailed operational flowchart of the integration process).

### 4.3. Phases of Implementation

**Figure 2:**
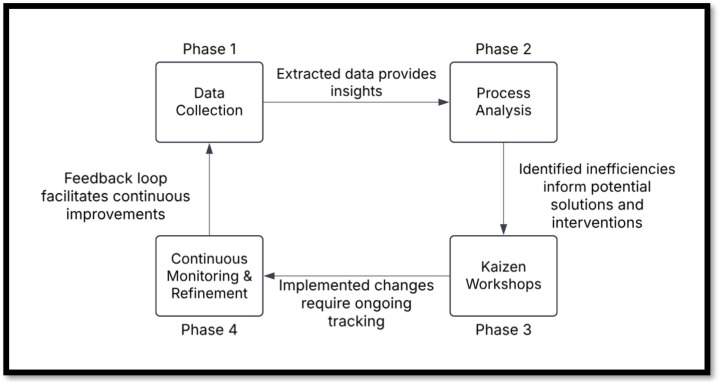
Phases of integrating Kaizen and Process Mining for continuous improvement in healthcare.

The integration of Kaizen and PM follows a structured approach to ensure sustainable and data-driven continuous improvement in healthcare settings. The implementation unfolds through four key phases, each addressing a critical component of the process. Figure 2 illustrates the four implementation phases, highlighting the iterative process of data collection, analysis, collaborative improvement, and continuous monitoring. To further clarify the practical application of these phases in healthcare environments, Table 2 provides concrete examples of activities and expected outputs for each implementation phase.

#### 4.3.1. Phase 1: Data Collection

The implementation begins with gathering event logs and process-related data from hospital systems. Electronic health records (EHRs), patient admission records, scheduling systems, and other operational data sources provide the foundation for PM analysis [85]. Ensuring data quality, completeness, and consistency is crucial at this stage, as inaccurate or incomplete event logs may lead to misleading insights. Standardized data extraction protocols help to streamline this process, enabling effective analysis in later phases [86]. The importance of meticulous data collection in healthcare is well documented. Research has shown that incomplete or inconsistent data negatively affect the ability to identify inefficiencies and workflow deviations [49,76]. Additionally, investigations into preoperative workflow analysis highlight the necessity of comprehensive data collection to enhance decision-making and improve process efficiency [87].

#### 4.3.2. Phase 2: Process Analysis

Once the event logs are collected, PM techniques are applied to uncover the actual workflow patterns. Discovery algorithms such as Alpha Miner (for structured processes) [88] and Heuristics Miner (for handling noise in complex healthcare workflows) [89] generate visual process models based on real-world execution. These visualizations reveal specific inefficiencies, including the following:

- Bottleneck analysis identifying critical pathway constraints (e.g., repetitive billing verification steps causing delays of up to 45 min in patient administrative processes) [90];
- Variant analysis showing deviations from standard care pathways (highlighting the most frequent alternative routes) [91];
- Performance indicators such as average case duration, processing times, and waiting times between activities;
- Social network analysis revealing handoff patterns between healthcare professionals [92].

Conformance checking techniques then compare these discovered workflows with the predefined standards, quantifying compliance gaps through fitness metrics and identifying specific points of deviation [93,94]. For example, this technique has been applied to assess adherence to clinical guidelines, ensuring that actual practices align with the established protocols [59]. Process enhancement algorithms identify opportunities for workflow optimization by analyzing temporal patterns and resource allocation efficiency [83].

The outputs from this phase—visual process maps, bottleneck identification, compliance metrics, and resource utilization analyses—serve as an objective basis for process optimization, providing healthcare teams with data-driven evidence to target specific improvement initiatives.

#### 4.3.3. Phase 3: Kaizen Events

The insights derived from PM feed directly into KEs, where healthcare teams collaboratively analyze inefficiencies and develop targeted improvement strategies. In these sessions or events, cross-functional teams engage in structured problem-solving using Kaizen tools such as root cause analysis (RCA), brainstorming, and value stream mapping (VSM). The PDSA/PDCA cycles provide a methodological framework to systematically test and implement changes.

Employee involvement at this stage serves multiple critical functions: it ensures that process changes are practical and aligned with operational realities, builds organizational commitment to improvement initiatives, and leverages frontline expertise in solution development, implementation, and sustainment. Research indicates that participation in continuous improvement correlates with enhanced perceptions of performance dimensions including quality, efficiency, and predictability [95]. Additionally, evidence suggests that employee engagement in healthcare settings positively impacts patient outcomes and safety metrics [96].

#### 4.3.4. Phase 4: Continuous Monitoring and Refinement

After implementing changes, the effectiveness of the interventions is continuously monitored using PM tools. Event logs are reanalyzed to assess whether the identified inefficiencies have been resolved and to detect any emerging issues. The feedback loop between Kaizen and PM ensures that improvements remain sustainable over time. A study has shown that integrating PM with continuous improvement methodologies allows for real-time analysis and the refinement of healthcare workflows, ensuring that process enhancements are both effective and enduring [97].

### 4.4. Implementation Considerations

Previous studies have indicated that continuous improvement initiatives can lead to staff resistance or fatigue if not accompanied by effective change management and engagement strategies [42,98]. These challenges reflect broader patterns identified in the Lean healthcare literature, particularly regarding the role of leadership, staff engagement, and system readiness for continuous improvement [99]. Therefore, the successful implementation of this framework requires robust change management strategies to mitigate resistance and prevent burnout among staff participating in continuous improvement efforts. Effective approaches include clearly communicating the purpose and expected benefits of process changes, transparently sharing Process Mining results to demonstrate value, recognizing staff contributions to improvement initiatives, scheduling dedicated time for improvement activities within normal workflows, and implementing changes gradually to prevent overwhelming staff with simultaneous process modifications [100,101,102]. Creating psychological safety is particularly crucial, allowing healthcare professionals to voice concerns about potential changes without fear of negative consequences [103,104]. Organizations should also incorporate regular assessments of staff well-being throughout the implementation process, adjusting the pace and scope of improvement initiatives accordingly [105,106,107].

### 4.5. Expected Outcomes

The integration of Kaizen and PM in healthcare aims to deliver significant improvements across multiple dimensions of healthcare operations. Table 3 presents the anticipated improvements following the application of this approach.

## 5. Validation and Application Scenarios

To ensure the effectiveness of the proposed integration of Kaizen and PM, the framework requires both conceptual validation and real-world application. This section outlines methodologies for validation, key performance indicators for assessment, and potential application scenarios in healthcare settings where this model could be implemented.

### 5.1. Validation Methodologies

The proposed framework can be validated using multiple approaches, including simulation models, pilot studies, and case studies. Table 4 represents the validation methodologies used to assess the effectiveness of the integrated Kaizen and PM framework in healthcare settings.

### 5.2. Metrics for Assessing Effectiveness

To measure the impact of the Kaizen–PM integration, various quantitative and qualitative metrics can be used:

**Operations/Process Outcomes**

- **Workflow Efficiency**: Metrics such as average patient wait times, time spent on administrative tasks, and service completion rates can indicate improvements in hospital operations [90,91];
- **Error Reduction:** A decrease in medication errors, scheduling conflicts, and process deviations would reflect the success of conformance checking and continuous process improvements [62];
- **Resource Utilization:** Comparing staff workload distribution, bed occupancy rates, and utilization before and after implementing the model can demonstrate optimization in resource allocation [122].

**Social Outcomes**

- **Patient Outcomes:** Patient feedback surveys and adherence to treatment protocols can serve as indicators of improved quality of care and patient experience [123];
- **Employee Outcomes:** Employee engagement and satisfaction are vital for sustaining continuous improvement efforts. Metrics such as workplace engagement levels, burnout and turnover rates, and participation in continuous improvement initiatives can provide insights into staff morale and the effectiveness of process changes [22].

**Clinical Outcomes**

- **Clinical Effectiveness:** Metrics such as reduced medical errors, reduced hospital readmission rates, fewer hospital-acquired conditions, and lower patient mortality and morbidity rates reflect the direct impact on patient health and safety [22].

**Financial Outcomes**

- **Cost and Utilization Metrics:** Operational cost savings, reduced average length of hospital stay, increased reimbursement efficiency, and cost-effectiveness analyses help to quantify the financial value of integrated Kaizen–PM initiatives [22].

### 5.3. Illustrative Application Scenarios

This framework can be applied in various real-world healthcare settings to optimize critical hospital processes:

1. **Emergency Department Operations:** PM can identify bottlenecks in triage, diagnostics, and patient transfers [124], while Kaizen workshops engage staff in refining these workflows [36];
2. **Patient Discharge Processes:** Delays in patient discharge contribute to overcrowding and inefficiencies. The framework could analyze event logs to detect administrative holdups [110], while Kaizen teams collaborate to streamline documentation, medication reconciliation, and discharge planning [125];
3. **Operating Room Scheduling:** PM can uncover inefficiencies in surgery scheduling and turnover times [112], and Kaizen interventions can introduce standardized best practices to ensure smoother transitions between surgical procedures [126].

## 6. Limitations and Future Research

The proposed integration of Kaizen and PM offers a promising framework for continuous improvement in healthcare. However, like any model, it has its limitations and challenges that warrant acknowledgment. These constraints serve as valuable opportunities for refinement and future exploration to ensure the framework’s success and adaptability in real-world applications.

### 6.1. Theoretical Constraints

The framework assumes the availability of high-quality, complete, and standardized event log data from hospital systems. However, healthcare data often suffer from inconsistencies [127], missing information [128], and a lack of uniformity [129], which can hinder the effectiveness of PM tools. Missing data can lead to incomplete process models that deviate from actual business processes and fail to capture critical workflow paths [130], while inconsistent timestamps may distort the perceived duration of activities and create artificial bottlenecks in the analysis, affecting performance metrics in healthcare settings [131,132]. Furthermore, incorrect event classifications can result in misleading conformance checking results [133], causing healthcare teams to address non-existent problems while overlooking actual inefficiencies, a challenge also reflected in healthcare event coding systems [134]. Additionally, the model relies on the assumption that insights derived from PM can seamlessly align with the human-driven improvement processes of Kaizen, which may not always hold true in complex and dynamic healthcare environments. Furthermore, the integration does not explicitly address the potential for variability in outcomes across different hospital settings, which may require context-specific adjustments to the framework.

### 6.2. Practical Challenges

Implementing the integration of Kaizen and PM in healthcare settings presents several practical challenges. Resource requirements, such as the need for skilled personnel, advanced PM tools, and time for Kaizen workshops, may strain already limited hospital resources [23]. Recent research by Munoz-Gama et al. has further documented that healthcare facilities without dedicated data analysts struggle to sustain integrated improvement approaches despite initial enthusiasm [23]. Staff resistance is another critical barrier, as healthcare employees may be reluctant to adopt new technologies or processes due to concerns about increased workload or disruption to established routines [135]. Previous studies found that this resistance is particularly pronounced among clinical specialists who perceive data-driven approaches as threats to professional autonomy [136,137]. Moreover, the initial costs of training staff, procuring PM software, and adapting workflows could deter healthcare organizations, particularly those operating in resource-limited environments [138]. Therefore, context-specific adaptations, such as tailored training programs, simplified data analytics solutions, and phased implementation strategies, may be necessary to accommodate these variations effectively. Bernardi et al. identified persistent data quality issues as a significant obstacle, noting that inconsistent data entry practices across departments compromise the ability to generate accurate process models [139]. Ensuring leadership buy-in and long-term commitment to the integration process is crucial for overcoming these barriers [140]. Engaged leaders foster a positive implementation climate by being approachable, involved in daily operations, and providing mentorship. This approach helps to overcome barriers and enhances the capacity for successful implementation [141].

### 6.3. Methodological Limitations

Our methodology exhibits several limitations that warrant acknowledgment. The heterogeneity of the databases used—ranging from healthcare-specific resources (PubMed) to broader aggregators (Google Scholar)—introduces variability in article visibility, indexing standards, and metadata availability [142,143]. This may have led to inconsistent retrieval patterns across different domains of the literature. Additionally, our approach to article selection, while carefully structured, necessarily involved the subjective assessment of relevance and quality rather than utilizing standardized grading tools. This subjective component could introduce selection bias, despite our efforts to maintain consistency in our evaluation process [144].

Furthermore, the challenges of deduplication across structurally diverse databases potentially affected our inclusion decisions, particularly for works indexed in multiple repositories with slight bibliographic variations [145,146]. The language restriction to English-language publications may have excluded valuable contributions from non-English speaking regions where both Kaizen and PM have been implemented [147,148]. These limitations should be considered when interpreting and applying our proposed framework in different healthcare contexts.

### 6.4. Opportunities for Future Research

To address these limitations and enhance the proposed framework, future research can focus on the following areas:

- Empirical Validation: Conducting pilot studies or case studies in diverse healthcare settings is essential to evaluate the practical implementation of the framework and its impact on workflow efficiency, error reduction, and patient outcomes [149];
- AI and Advanced Analytics Integration: Exploring the incorporation of artificial intelligence and machine learning techniques to augment PM capabilities, such as predictive modeling for resource allocation or real-time decision support in healthcare processes [150];
- Cross-Sector Applications: Investigating the adaptability of this framework beyond healthcare, such as in manufacturing, education, or logistics, to assess its versatility and broader applicability. PM has been applied in various domains, and its methodologies can be tailored to different sectors to improve process efficiency and effectiveness [151]. Additionally, Kaizen has demonstrated its applicability across various sectors and industries [152];
- Data Quality Improvement: Developing standardized methods for cleaning and structuring healthcare data is crucial to ensure compatibility with PM tools and maximize the accuracy of insights. A perspective article explored how PM can extract clinical insights from mobile health data and complement data-driven techniques like machine learning, emphasizing the importance of data quality in such analyses [153];
- Human-Centric Adaptations: Examining strategies to further integrate frontline staff input and enhance their engagement in data-driven improvement processes, ensuring that the framework remains both actionable and practical [149].

## 7. Conclusions

Healthcare systems worldwide face increasing pressure to improve efficiency, optimize resource allocation, and deliver high-quality patient care. This paper proposes an integrated framework that combines the structured, collaborative problem-solving of Kaizen with the objective, data-driven insights of PM to address these challenges. The proposed model bridges the gap between traditional continuous improvement methodologies and modern analytical tools, enabling healthcare organizations to identify inefficiencies, reduce errors, and streamline workflows with greater precision. Frontline staff actively contribute to decision-making, ensuring that solutions align with the practical realities of healthcare operations and foster a culture of continuous improvement. The framework promises significant benefits, such as enhanced workflow efficiency, optimized resource utilization, and better patient outcomes, though challenges like data quality issues, resource constraints, and staff resistance remain. Future research should focus on empirical validation, AI-driven advancements, and cross-sector applications to refine and broaden the framework’s impact. This integration of Kaizen and PM offers a scalable, adaptable, and patient-centered approach to transforming healthcare process improvement.

## Figures and Tables

**Table 1:** Kaizen tools and methods for continuous improvement in healthcare.

Tool/Method	Description
**Iterative Improvement Cycles**	
Plan-Do-Study-Act (PDSA) Cycle Plan-Do-Check-Act (PDCA) Cycle	A structured, iterative approach to testing and implementing changes [73].
**Collaborative Problem-Solving Tools**	
Brainstorming Sessions	Collaborative problem-solving methods to generate creative and actionable solutions [74].
**Process Visualization Tools**	
Value Stream Mapping (VSM)	A technique for visualizing workflows to identify inefficiencies and areas for improvement [75].
Root Cause Analysis (RCA)	A systematic approach to identifying and addressing the underlying causes of issues [76].

**Table 2:** Phases of the integrated framework with example activities and expected outputs.

Phase	Example Activities	Expected Outputs
**Data Collection**	- Extract event logs from hospital EHR systems - Capture timestamp data from admission to discharge - Document process variations across units	- Structured datasets of patient flow events - Standardized event logs for analysis
**Process Analysis**	- Apply discovery algorithms to identify actual workflows - Perform conformance checking against protocols - Visualize bottlenecks in patient flow	- Process models of real workflows - Deviation reports highlighting inefficiencies - Delay pattern metrics
**Kaizen Events**	- Conduct cross-functional improvement workshops - Apply root cause analysis to bottlenecks - Implement PDSA/PDCA cycles for discharge streamlining	- Staff-driven improvement proposals - Standardized protocols for transitions - Implementation plans
**Continuous Monitoring**	- Reanalyze logs post intervention - Track key performance indicators (KPIs) - Identify emerging issues for new cycles	- Comparative analysis of intervention impact - Updated process models - Tracking and documentation of sustained gains

**Table 3:** Expected enhancements from the integration of Kaizen and Process Mining in healthcare.

Outcome Category	Process Mining Contribution	Kaizen Contribution
**Operational/Process Outcomes**		
**Streamlined Workflows**	Provides real-time visibility of operational inefficiencies and deviations from expected workflows [63].	Uses tools like value stream mapping (VSM) and PDCA cycles to systematically remove inefficiencies and standardize best practices [108].
Reduced Wait Times	Identifies bottlenecks by analyzing event logs in emergency departments, outpatient services, and surgical scheduling [14].	Implements staff reallocation, process streamlining, and workflow redesign to optimize patient flow [109].
Improved Decision-Making	Analyzes historical and real-time event logs to provide actionable insights for clinical adjustments [55].	Engages clinicians in continuous improvement discussions, ensuring that data-driven changes are clinically relevant [110].
**Clinical Outcomes**		
**Enhanced Compliance**	Uses conformance checking to compare actual workflows with predefined clinical protocols, identifying deviations [46].	Encourages staff accountability and proactive process refinement to align with evidence-based practices [32].
Better Safety and Quality	Identifies patterns and risks in care processes before they affect patients [110].	Establishes standardized protocols that reduce variation and enhance care reliability [111].
**Employee Outcomes**		
Cultivating **Improvement** Culture	Supplies objective, data-backed evidence to guide iterative improvements [112].	Actively involves frontline staff in problem-solving and process optimization, fostering ownership [113].
Enhanced Skills Development	Provides learning opportunities through data visualization and analysis [60].	Builds problem-solving capabilities through structured improvement approaches [114].
**Patient Outcomes**		
**Improved** Experience	Ensures smoother workflows and timely interventions through automated process monitoring [14].	Enhances service quality and patient-centered care by reducing inefficiencies [113].
**Financial Outcomes**		
Cost Reduction	Identifies resource waste and unnecessary process steps through detailed activity analysis [115].	Implements targeted efficiency improvements that reduce operational costs while maintaining quality [116].
Revenue Enhancement	Uncovers opportunities for optimizing reimbursement through analysis of billing processes and claim patterns [90].	Develops standardized approaches to documentation and coding that maximize appropriate revenue capture [117].

**Table 4:** Validation methodologies for the integration of Kaizen and PM in healthcare.

Validation Method	Description	Expected Benefits
Simulation-Based Validation	Computational simulations replicate hospital workflows to evaluate the integration of PM and Kaizen improvements [118].	Predict potential efficiency gains before real-world implementation [119].
Pilot Studies	Small-scale implementations in specific departments to test the framework in a controlled setting [35].	Identify challenges, refine the integration, and assess feasibility [120].
Case Studies	Reviewing past applications of Kaizen and PM in healthcare settings to conceptualize their combined impact [121].	Provide empirical insights into real-world effectiveness [23,89].

## Data Availability

The original contributions presented in this study are included in the article. Further inquiries can be directed to the corresponding author(s).

## Supplementary Materials

The following supporting information can be downloaded at: https://www.mdpi.com/article/10.3390/healthcare13080941/s1, Figure S1: Operational flowchart: Integrating Process Mining with Kaizen in Healthcare.
