# Supplementary figures and images for: Leveraging Kaizen with Process Mining in Healthcare Settings: A Conceptual Framework for Data-Driven Continuous Improvement
Source: Healthcare (Basel). 2025 Apr 19;13(8):941. doi: 10.3390/healthcare13080941 (PMC12026918; doi:10.3390/healthcare13080941)

Figure S1: Operational flowchart: Integrating Process Mining with Kaizen in Healthcare.

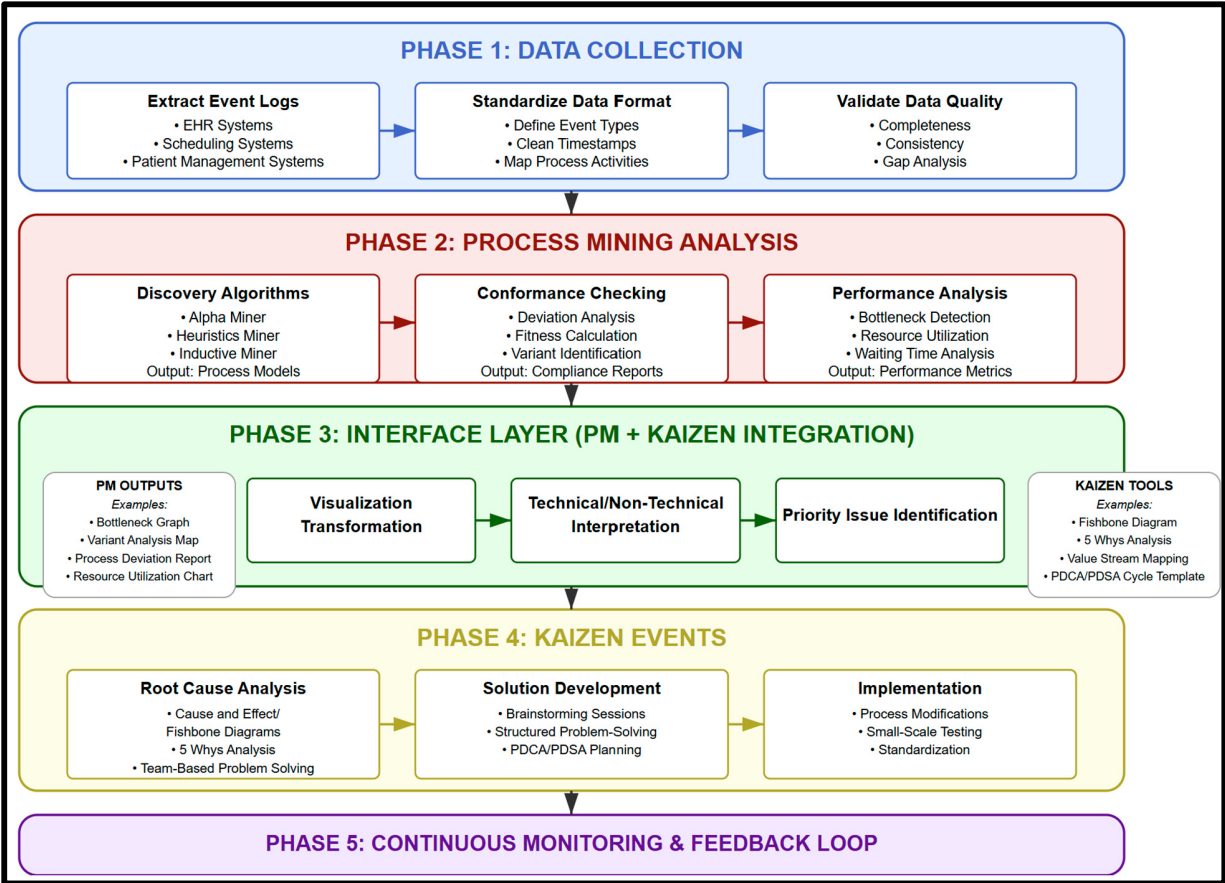

Supplement: Supplementary file 1 [file healthcare-13-00941-s001.zip › healthcare-3518259-supplementary.pdf]
